# Cetirizine and Levetiracetam as Inhibitors of Monoacylglycerol Lipase: Investigating Their Repurposing Potential as Novel Osteoarthritic Pain Therapies

**DOI:** 10.3390/ph16111563

**Published:** 2023-11-06

**Authors:** Corina Andrei, Dragos Paul Mihai, Georgiana Nitulescu, Anca Ungurianu, Denisa Marilena Margina, George Mihai Nitulescu, Octavian Tudorel Olaru, Radu Mihai Busca, Anca Zanfirescu

**Affiliations:** 1 Faculty of Pharmacy, “Carol Davila” University of Medicine and Pharmacy, Traian Vuia 6, 020956 Bucharest, Romania; 2 Colentina Clinical Hospital, Stefan cel Mare 19-21, 020125 Bucharest, Romania

**Keywords:** MAGL inhibitors, cetirizine, levetiracetam, osteoarthritis, analgesia, complete Freund’s adjuvant, molecular docking, inflammatory pain, drug repositioning

## Abstract

Osteoarthritis is characterized by progressive articular cartilage degradation, subchondral bone changes, and synovial inflammation, and affects various joints, causing pain and disability. Current osteoarthritis therapies, primarily focused on pain management, face limitations due to limited effectiveness and high risks of adverse effects. Safer and more effective treatments are urgently needed. Considering that the endocannabinoid 2-arachidonoyl glycerol is involved in pain processing, increasing its concentration through monoacylglycerol lipase (MAGL) inhibition reduces pain in various animal models. Furthermore, drug repurposing approaches leverage established drug safety profiles, presenting a cost-effective route to accelerate clinical application. To this end, cetirizine and levetiracetam were examined for their MAGL inhibitory effects. In vitro studies revealed that cetirizine and levetiracetam inhibited MAGL with IC_50_ values of 9.3931 µM and 3.0095 µM, respectively. In vivo experiments demonstrated that cetirizine, and to a lesser extent levetiracetam, reduced mechanical and thermal nociception in complete Freund adjuvant (CFA)-induced osteoarthritis in rats. Cetirizine exhibited a notable anti-inflammatory effect, reducing CFA-induced inflammation, as well as the inflammatory infiltrate and granuloma formation in the affected paw. These findings suggest that cetirizine may serve as a promising starting point for the development of novel compounds for osteoarthritis treatment, addressing both pain and inflammation.

## 1. Introduction

Osteoarthritis is a degenerative joint disease characterized by the progressive degradation of articular cartilage, subchondral bone changes, and synovial inflammation, primarily affecting weight-bearing joints such as the knees, hips, and hands. It leads to debilitating pain, stiffness, joint deformities, and reduced range of motion, ultimately impairing an individual’s physical function and quality of life [1]. Osteoarthritis poses a substantial societal and economic burden, contributing to disability and imposing significant healthcare costs due to the need for treatments, joint replacements, and rehabilitative services for affected patients, underscoring the pressing need for effective management strategies and therapies [2]. The therapeutic options for osteoarthritis, primarily aimed at managing its debilitating pain, face challenges as current analgesics including anti-inflammatory drugs (NSAIDs) or opioids have limited efficacy and carry a high risk of adverse effects. Intra-articular corticosteroid injections have limited long-term efficacy and may contribute to joint deterioration [3]. Consequently, there is a growing need to identify safer and more effective treatments to address the pain burden in osteoarthritis patients with improved outcomes and fewer side effects.

2-Arachidonoylglycerol (2-AG) is an endocannabinoid lipid molecule that plays a pivotal role in the endocannabinoid system, a complex regulatory network in the human body. It is synthesized on demand from phospholipid precursors and acts as a partial agonist of cannabinoid receptors, primarily CB1 and CB2 [4]. This bioactive lipid is involved in various physiological processes, including pain perception, inflammation, immune response, appetite regulation, and synaptic plasticity and transmission, making it a crucial mediator in maintaining homeostasis within the body. Furthermore, disturbances in 2-AG metabolism have been observed in osteoarthritic joints, where it acts as a signaling molecule through cannabinoid receptors, particularly CB1 and CB2. By binding to these receptors, 2-AG can influence pain perception, attenuate inflammation, and potentially regulate chondrocyte activity and cartilage degradation [5]. These findings suggest that 2-AG may have a regulatory function in osteoarthritis progression. Furthermore, 2-AG is hydrolyzed to arachidonic acid and glycerol by monoacylglycerol lipase (MAGL). Inhibiting MAGL could potentially modulate 2-AG levels, reducing osteoarthritis-associated pain and inflammation [6]. Therefore, MAGL inhibition represents a novel therapeutic strategy with the potential to mitigate osteoarthritis symptoms and improve patient outcomes. Furthermore, a drug repurposing campaign aimed at identifying novel MAGL inhibitors for osteoarthritis treatment would provide a cost-effective and efficient approach, harnessing existing drugs that have established safety profiles and potentially accelerating the path to clinical application.

To this end, considering our preliminary results based on structure-activity relationship analysis of known MAGL inhibitors and toxicity-based screening of approved drugs (data not shown), we selected cetirizine, an H1 receptor antagonist approved for use as an antiallergic, and levetiracetam, an antiepileptic known to target neuronal synaptic vesicle protein 2A, to be assessed as potential MAGL inhibitors. After MAGL inhibitory activity was demonstrated in vitro, we tested the analgesic and anti-inflammatory effects of these compounds using the complete Freund adjuvant (CFA)-induced osteoarthritis model in rats. Moreover, we used molecular docking approaches to provide a deeper insight into their interaction with MAGL.

## 2. Results

### 2.1. MAGL Activity Assay

**Figure 1:**
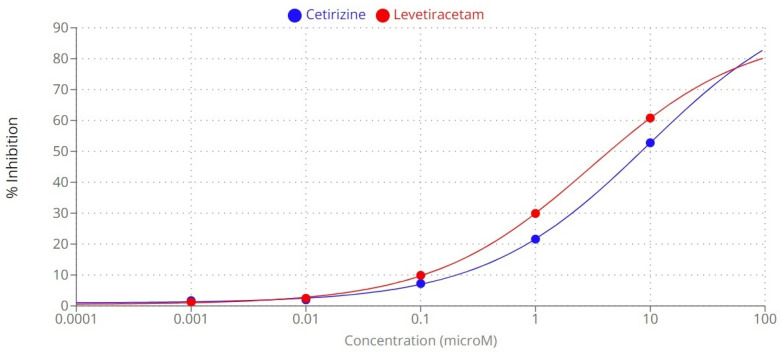
The inhibitory effect of levetiracetam and cetirizine on monoacylglycerol lipase after incubation.

Levetiracetam and cetirizine were tested at various concentrations to evaluate their inhibitory effect on MAGL (Figure 1). The IC_50_ values were calculated for both compounds after incubation (Table 1). Based on our assay, levetiracetam was more potent (3.0095 µM) than cetirizine (9.3931 µM) in inhibiting MAGL, while the positive control (JZL 195) had a potency of 8 nM. Even though the IC_50_ values for cetirizine and levetiracetam are in the micromolar range, previous studies indicated that antinociceptive activities can be exerted by the triterpene β-amyrin via inhibition of MAGL at IC_50_ values of 2.800 ± 0.500 µM, while the derivative α-amyrin had a potency of 9.300 ± 1.200 µM [7].

### 2.2. Molecular Docking

**Figure 2:**
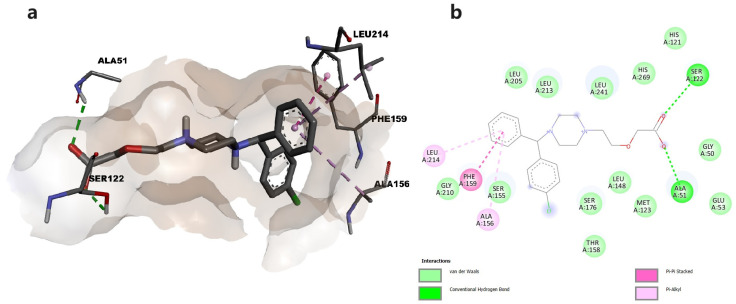
(**a**) Docked conformation of cetirizine and predicted interactions with MAGL; (**b**) predicted interactions represented in 2D diagram in which ligand-protein interactions are colored depending on their type: conventional hydrogen bonds in green, π-σ in purple, and π-alkyl interactions in light purple, respectively.

To better understand the interaction between the two tested compounds and MAGL, we performed a molecular docking study. Thus, we predicted the binding affinities between cetirizine or levetiracetam and MAGL, and the interactions that could be responsible for enzymatic inhibition. The piperazinyl moiety of cetirizine functions as a linker between the aromatic groups located in the hydrophobic portion of the protein and the carboxylic group, located in the oxoanionic channel of the enzyme, where the catalytic triad is located. Thus, through the oxygen atoms in the carboxylic group, it forms hydrogen bonds with Ser122, one key residue within the active site of the enzyme, and Ala51. The phenyl group occupies the hydrophobic cavity and forms π-π and π-alkyl interactions with the corresponding hydrophobic amino acids (Figure 2).

**Figure 3:**
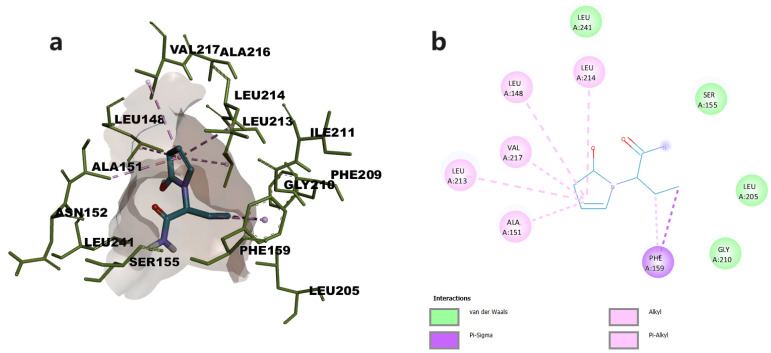
(**a**) Docked conformation of levetiracetam and predicted interactions with MAGL; (**b**) predicted interactions represented in 2D diagram in which ligand-protein interactions are colored depending on their type: π-σ interactions in purple, and π-alkyl interactions in light purple, respectively.

The oxopyrrolidine core of levetiracetam was predicted to bind in the open hydrophobic cavity of the enzyme, the complex being stabilized through hydrophobic π-π and π-alkyl interactions with residues Val 217, Leu213, Leu214, Leu148, and Ala151. Levetiracetam also established van der Waals contacts with various residues including Ser122 (Figure 3).

### 2.3. Mechanical Pain Response

Statistically significant variations were observed on day 7 (Kruskal-Wallis, H = 14.28, *p* = 0.0139). CFA treatment significantly decreased the mechanical pain threshold, with a maximum effect observed on day 7 (*p* < 0.01, Dunn post hoc, Table 2). Hyperalgesia induced by CFA was counteracted by the administration of tramadol, the reference analgesic. Thus, tramadol-treated animals showed mechanical sensitivity similar to that of the CTL group. Cetirizine partially alleviated the CFA-induced mechanical hyperalgesia (*p* < 0.05), but its effectiveness was lower than that of tramadol. Even though levetiracetam also reduced mechanical hyperalgesia in CFA-injected rats, the effect was not statistically significant (*p* > 0.05).

### 2.4. Thermal Pain Response

Significant changes in thermal sensitivity were observed between groups after 14 days of treatment (univariate ANOVA, F = 19.10, *p* < 0.0001). Intraplantar administration of CFA caused an increase in the thermal sensitivity threshold when compared to both baseline values and control group (Table 3). Thermal hypersensitivity increased in intensity up to day 14 (*p* < 0.05, Bonferroni correction) and was mitigated by the administration of tramadol (*p* < 0.01) and partially by that of cetirizine (*p* < 0.05). However, the antinociceptive effects of levetiracetam were modest (*p* > 0.05).

### 2.5. Anti-Inflammatory Effect

**Figure 4:**
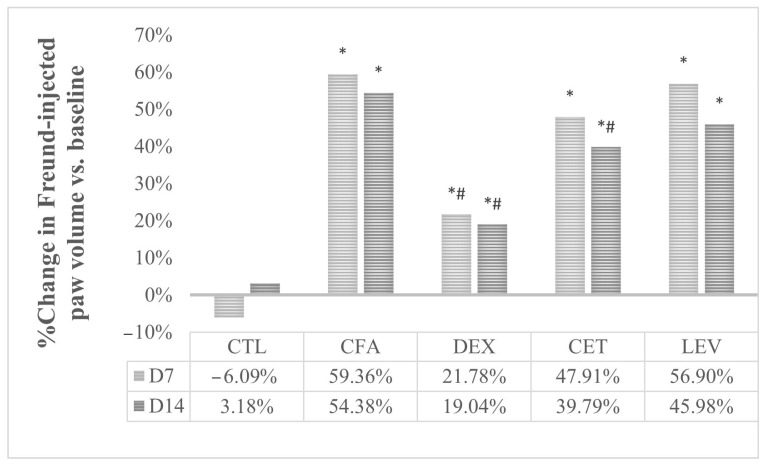
Evolution of paw edema in comparison with baseline values. CTL, control group; CFA, complete Freund adjuvant-treated group; CFA + DEXA, complete Freund adjuvant and dexamethasone-treated group; CFA + LEV, complete Freund adjuvant and levetiracetam-treated group; CFA + CET, complete Freund adjuvant and cetirizine-treated group; * *p* < 0.05 vs. CTL; ^#^ *p* < 0.05 vs. CFA.

Significant changes were observed in percentage variation of paw volume after 7 (univariate ANOVA, F = 14.01, *p* < 0.05) and 14 days (Kruskal-Wallis, H = 30.68, *p* < 0.05) of treatment. CFA-injected rats exhibited a significant increase in the volume of the ipsilateral hind paw compared to the control (*p* < 0.05, Bonferroni correction). No significant changes in paw volume were observed in control rats. As illustrated in Figure 4, dexamethasone significantly inhibited the CFA-induced edema (*p* < 0.05, Bonferroni correction). Additionally, cetirizine also alleviated inflammation induced by CFA, the effect reaching statistical significance on day 14 (*p* < 0.05). No anti-inflammatory effect was observed for levetiracetam.

### 2.6. Histopathological Analysis

**Figure 5:**
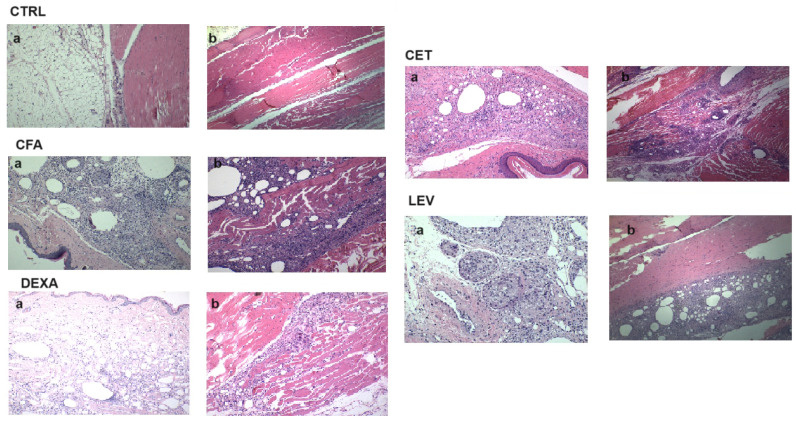
Histological section in the sagittal plane (10X/20X) of hind paw tissues in experimental animals. Representative micrographs of hematoxylin and eosin (H&E) stained sections of rat paws in each group on the 18th day of complete Freund’s adjuvant-induced arthritis with an enlargement ratio of 200 μm: (**a**) subcutaneous tissue; (**b**) muscle tissue.

The histopathological analysis (Figure 5) revealed no tissue abnormality in the control group. The CFA-treated group presented an inflammatory infiltrate consisting of lymphocytes, plasma cells, macrophages, or epithelioid histiocytes with granuloma formation (granulomatous inflammation) and multinucleated, polymorphonuclear neutrophilic cells. Based on the degree of inflammation, qualitative scores were given for each tested group (univariate ANOVA, F = 47.93, *p* < 0.0001). Inflammatory cell infiltration was significantly increased by CFA injection (*p* < 0.001, Bonferroni correction) and inhibited by dexamethasone treatment (*p* < 0.01, Table 4). A similar trend was observed for cetirizine-treated rats, but the effect was not statistically significant (*p* > 0.05).

## 3. Discussion

The present work aimed to investigate the repurposing potential of levetiracetam and cetirizine in osteoarthritic pain. Both approved drugs have acceptable safety profiles and are well tolerated by patients [3]. The most common adverse effects associated with levetiracetam and cetirizine are shown in Table 5.

Our in vitro studies revealed that cetirizine and levetiracetam inhibited MAGL with potencies in the micromolar ranges, showing IC_50_ values of 9.3931 µM and 3.0095 µM, respectively. Specific structural features such as the piperazine moiety connected to a carboxyl group within cetirizine and the pyrrolidinone moiety in levetiracetam were previously shown to contribute to MAGL inhibitory activities [10]. According to the performed molecular docking study, the carboxylic group of cetirizine establishes a hydrogen bond with Ser122, one of the three amino acids in the catalytical site of the enzyme, while levetiracetam was involved only in hydrophobic interactions. In the presence of the endogenous substrate, 2-AG, Ser122 initiates a nucleophilic attack on the carbonyl of the substrate, triggering the enzymatic transformation [11].

The in vivo studies confirmed our hypothesis for cetirizine, and to a lesser extent for levetiracetam, highlighting increased mechanical and thermal pain thresholds in CFA-treated rats. Previous studies reported analgesic effects of cetirizine at 1 mg/kg in mice using three pain models—tail flick, tail immersion, and tail clip [12]. Furthermore, cetirizine administered intraperitoneally at 20 mg/kg significantly inhibited acute trigeminal pain in rats [13,14]. Locally administered at doses of 0.25 and 1 µmol/paw, cetirizine exhibited an analgesic effect in the second phase of formalin-induced pain. The formalin test comprises two phases with different nociceptive mechanisms: the early phase results from a direct nociceptor effect without significant involvement of prostaglandins, while the late phase is associated with inflammatory pain [15].

Previous studies showed that levetiracetam (200–1000 nmol/paw) demonstrated an analgesic effect in rats with carrageenan-induced inflammation. However, it did not influence the thermal pain threshold in the contralateral normal paw [16]. In humans, levetiracetam might provide some relief in neuropathic and multiple sclerosis-associated pain [17], though definitive conclusions are challenging due to study designs. Both levetiracetam and cetirizine potentiate the analgesic effect of various analgesics, including morphine [18], acetaminophen, and nonsteroidal anti-inflammatory drugs [19]. Our data further support these results, revealing cetirizine as an effective analgesic in osteoarthritis, and MAGL inhibition as a mechanism underlying at least in part the analgesic effects of the tested compounds. MAGL inhibitors were shown to exhibit analgesic effects in various pain models. MAGL inhibitor JZL 184 at 5, 10, and 20 mg/kg elicited a dose-dependent analgesic effect in inflammatory visceral pain [20] through a CB1 receptor-mediated mechanism of action. Similar results were reported using inflammatory, neuropathic, thermal, and chemically-induced pain models [21,22,23].

The higher analgesic efficacy of cetirizine in comparison with levetiracetam may be explained by its effect on the histaminergic receptors. Histamine has a dual role in nociceptive response processing. It has both a central analgesic effect, mediated by the activation of H4 receptors, and a pro-nociceptive effect, mediated by the activation of H1, H2, and H3 receptors [24]. Histamine is involved in the peripheral stimulation of nociceptive fibers, and its antagonists, even for those that do not cross the blood-brain barrier, have analgesic effects [25]. Therefore, H1 receptor blockage by cetirizine could also contribute to its analgesic effect.

Cetirizine, but not levetiracetam, exhibited a substantial anti-inflammatory activity. The H1 antagonist reduced CFA-induced inflammatory infiltrate and granuloma formation in the subcutaneous and muscular tissues of the injected paw, changes that accompany the degenerative process in osteoarthritis [1]. These results suggest that other mechanisms could also contribute to the observed anti-inflammatory effect. This hypothesis remains to be demonstrated in further studies. The H1 blocking activity might be at least partly responsible for the anti-edematous effect. In vitro studies have shown that histamine stimulates the production of degrading enzymes and inflammatory mediators associated with degenerative joint disease in synovial fibroblasts and human articular chondrocytes [26,27]. Additionally, histamine induces KIAA1199 (cell migration inducing hyaluronidase 1) expression in fibroblasts [28]. KIAA1199 regulates the depolymerization of hyaluronan, which is crucial for overall joint health [28].

Our study comprises several limitations. First, we did not delve into the specific type of enzymatic inhibition exhibited by cetirizine and levetiracetam on MAGL, thus it is unclear whether the two drugs are competitive or noncompetitive (allosteric) inhibitors. This area could benefit from further exploration using kinetic studies and biophysical methods, as it may provide valuable insights into protein–ligand interaction strength (K_d_ values) and the precise mechanism by which these compounds exert their analgesic effects. Furthermore, we have not conducted experiments aimed at elucidating the exact extent of MAGL inhibition contribution to the analgesic properties of the tested compounds, with a particular focus on cetirizine. Further studies could investigate the analgesic and anti-inflammatory activities of cetirizine in combination with cannabinoid receptors antagonists/inverse agonists to understand the relative importance of endocannabinoid signaling pathway in the overall analgesic mechanism of the repurposing candidate, while the enzymatic activity of MAGL could also be assessed in tissue samples at the end of the experiments. Moreover, follow-up investigations could be performed to compare the anti-inflammatory and analgesic effects of cetirizine with well-known MAGL inhibitors (e.g., pristimerin [29]) and other H1 receptor blockers that lack MAGL inhibitory activity to gain more insight into the in vivo therapeutic efficacy. Lastly, multiple doses of levetiracetam and cetirizine could be assessed as single therapies and in combination with approved anti-inflammatory and analgesic drugs to establish ED_50_ (median effective dose) values and their synergistic effects, since such associations could lower both the therapeutically effective doses and the risks of adverse events. These future experiments could shed more light on cetirizine’s therapeutic potential as an analgesic and anti-inflammatory agent and help refine its clinical applications. Addressing these aspects in future research could provide a more comprehensive understanding of the therapeutic potential of these compounds.

Another potential application of levetiracetam and cetirizine as MAGL inhibitors would be cancer treatment. Previous studies highlighted that MAGL inhibition may be beneficial in managing prostate, gynecological and colorectal cancers [30]. Moreover, MAGL inhibitor JJKK048 was shown to reduce hypoxia-induced resistance to multi-kinase inhibitor regorafenib in triple negative breast cancer cells by downregulating ABCG2 transporter expression, and thus reducing the cellular export of the anticancer drug [31]. Interestingly, another study showed that MAGL inhibition can exert antiangiogenic effect by inducing the release of tissue inhibitor of metalloproteinase-1 (TIMP-1) from lung cancer cells [32].

## 4. Materials and Methods

### 4.1. MAGL Activity Assay

All reagents and solvents were purchased from Sigma-Aldrich. The in vitro direct inhibition of the MAGL enzyme was measured using a colorimetric procedure based on a commercial kit (CAY705192, Cayman Chemical, Ann Arbor, MI, USA). MAGL hydrolyzes 4-nitrophenyl acetate resulting in 4-nitrophenol, a yellow product. Five different concentrations (0.001, 0.01, 0.1, 1, and 10 μM) of each test compound were tested. JZL 195 (CAS: 1210004-12-8), provided by Cayman, was used as reference inhibitor (positive control), while the vehicle (DMSO) was used as a negative control. The experiment was performed as per manufacturers guidelines. Absorbance was read at 405 nm on Varioskan™ LUX multimode microplate reader (ThermoFisher Scientific Inc., Waltham, MA, USA). The experiment was performed in duplicate. The inhibition (I%) values were calculated and plotted against the logarithm of concentrations. The half-maximal inhibitory concentration (IC_50_) was calculated using an online tool: MLA “Quest Graph™ IC_50_ Calculator” (AAT Bioquest, Inc., https://www.aatbio.com/tools/ic50-calculator accessed on 1 September 2023).

### 4.2. Animals

Sprague–Dawley male rats (*n* = 60) were purchased from the biobase of INCDMI Cantacuzino, Bucharest, Romania, and acclimated to laboratory conditions for 1 week prior to experiments. They were housed ten per cage, at 24–30 °C and 60–70% humidity, and had free access to water and food with 12-h light/dark cycle. All procedures used in this study were designed in accordance with ARRIVE guidelines and NIH Guide for the Care and Use of Laboratory Animals. The experimental protocol was approved by the Ethics Committee of the University of Medicine and Pharmacy, Bucharest (no. 0039/2019).

### 4.3. Induction of Osteoarthritis

Rats were randomly divided into six groups (*n* = 10). Osteoarthritis was induced by a single intradermal injection of 0.1 mL of CFA (Sigma) into the right hind paw of experimental [33] groups, except for the control (CTL), which was injected with paraffin oil 0.1 mL/paw. After osteoarthritis induction, animals received the following treatments: CTL, distilled water p.o. 1 mL/100 g; CFA, distilled water p.o. 1 mL/100 g; CFA + CET, cetirizine 10 mg/kg p.o; CFA + DEX, dexamethasone (reference for anti-inflammatory activity), 0.5 mg/kg p.o.; CFA + TRM, tramadol (reference for analgesic effect), 80 mg/kg p.o.; CFA + LEV 330 mg/kg p.o. The duration of the experiment was 18 days, after which the animals were sacrificed using cervical dislocation, and the right hind paw was collected and processed for histological analysis.

### 4.4. Randall–Selitto Test

The Randall–Selitto test evaluates hyperalgesia induced by increasing paw pressure using an analgesy-meter (Ugo Basile, 37215, Verase, Italy). The right hind paws were exposed to increasing force until vocalization was achieved or until maximal force (250 g) was obtained, whichever occurred first [34,35,36].

### 4.5. Hot Plate Test

The Hot Plate test was performed as previously described [37]. Mice were placed on a hot plate (Ugo Basile Biological Research Apparatus, Varese, Italy), with the temperature adjusted to 53 ± 0.1 °C. We evaluated the time to the first sign of nociception (paw licking) before (baseline latency) and after the administered treatments. After exhibiting this behavior, mice were immediately removed from the Hot Plate. An arbitrary cutoff period of 45 s was adopted to avoid damage to the paws.

### 4.6. Paw Volume Assessment

Rat paw volume was measured using a plethysmometer (Ugo Basile Plethysmometer 7140) with the following device specifications: water tanks: 3.5 cm for rats; device resolution: 0.01 mL. The edema was quantified by measuring the differences in the paw volume between the day 0 and other different time points of the study—day 7 and day 14 after the induction of the osteoarthritis model [38].

### 4.7. Histological Assessment

Hind paws were removed by cutting at a distance of 2–3 mm above the ankle. Sample preparation, decalcification, and staining procedures were performed according to ‘SMASH’ recommendations for standardized microscopic arthritis scoring of histological sections from inflammatory arthritis animal models [39]. In this experiment we used standard hematoxylin–eosin staining for the evaluation of the degree of inflammation and the distribution of cells in the tissues. Images of posterior paw sections were performed using a light microscope (Euromex bScope, Euromex Microscopen BV, Papenkamp, The Netherlands) and digital camera (C-mount camera, Carl Roth GmbH + Co. KG, Karlsruhe, Germany). The degree of inflammation was determined using a score ranging from 0 to 3 as follows: 0—no inflammatory infiltrate; 1—minimal inflammatory infiltrate; 2—moderate, diffuse inflammatory infiltrate; 3—severe, diffuse inflammatory infiltrate [39].

### 4.8. Molecular Docking

The crystal structure of human MAGL (5ZUN [40]) was obtained from the RCSB PDB database and the inhibitor 9JX was obtained from PubChem. Structures were optimized using YASARA software (http://www.yasara.org/ accessed on 1 September 2023) [41], by performing energy minimization with AMBER03 forcefield and protonation according to physiological pH [42]. The co-crystallized ligand was removed and then docked into the active site to validate the predicted conformation of the complex. Three-dimensional structures of the cetirizine and levetiracetam were retrieved from PubChem. Ligands were minimized using MMFF94s + forcefield, protonated at physiological pH, and docked within the active site of MAGL using AutoDock Vina v1.1.2 within YASARA [43]. Twenty-five docking runs were performed for each ligand. Docking results were retrieved as the binding energy (ΔG, kcal/mol) of the best binding pose of both ligands. The conformations of the predicted protein–ligand complexes and molecular interactions were analyzed using BIOVIA Discovery Studio Visualizer (BIOVIA, Discovery Studio Visualizer, Version 17.2.0, Dassault Systèmes, 2016, San Diego, CA, USA).

### 4.9. Statistical Analysis

Statistical analysis was performed using GraphPad Prism version 5 software (GraphPad Software, Inc., La Jolla, CA, USA). Student’s *t*-test, one-way ANOVA (followed by Tukey’s test for post hoc comparison), or two-way ANOVA (followed by Bonferroni test for post hoc analysis) were employed for data analysis. Data are expressed as mean ± mean standard error. *p* < 0.05 were considered statistically significant.

## 5. Conclusions

In silico, in vitro and in vivo studies were carried out to investigate the repurposing potential of levetiracetam and cetirizine as novel therapeutic solutions for managing osteoarthritic pain. Our data suggests that the multitarget compound cetirizine—an inhibitor of MAGL and H1 antagonist—might be an effective therapeutic approach in osteoarthritis, reducing both pain, the central symptom of the disease, and inflammation, a contributor to tissular degeneration. Therefore, cetirizine might serve as a starting point for the development of such compounds.

## Figures and Tables

**Table 1:** The inhibitory effect of tested compounds on MAGL.

Compound	IC_50_ (µM)	Concentration (µM)	Inhibition (%, mean ± S.E.M.)
Cetirizine	9.3931	10	52.775 ± 0.925
		1	21.610 ± 1.859
		0.1	7.230 ± 1.049
		0.01	1.975 ± 5.615
		0.001	1.690 ± 3.140
Levetiracetam	3.0095	10	60.800 ± 2.599
		1	29.920 ± 0.509
		0.1	9.880 ± 1.309
		0.01	2.455 ± 0.304
		0.001	1.225 ± 0.214

IC_50_, half-maximal inhibitory concentration; S.E.M., standard error of the mean.

**Table 2:** Mechanical pain threshold for tested groups.

Rat	CTL			CFA			CFA + TRM			CFA + LEV			CFA + CET		
	Day 0	Day 7	Day 14	Day 0	Day 7	Day 14	Day 0	Day 7	Day 14	Day 0	Day 7	Day 14	Day 0	Day 7	Day 14
Mean ± S.E.M	22.50 ± 1.57	21.15 ± 1.71	21.90 ± 1.49	23.15 ± 1.26	12.24 ± 2.08	17.65 ± 2.18	21.70 ± 2.00	20.45 ± 2.05	22.35 ± 1.64	24.95 ± 0.05	16.56 ± 2.36	19.39 ± 1.73	25.00 ± 0.00	19.30 ± 1.78	21.90 ± 1.49
% variation vs. baseline		−6	−2.67		−47.13	−23.76		−5.76	2.99		−33.63	−22.28		−22.8	−12.4
% variation vs. control					−41.13 **	−21.09		0.24	5.66		−27.63	−19.62		−16.8	−9.73
% variation vs. CFA								41.37 ^#^	26.75		13.50	1.47		24.33 ^#^	14.02

CTL, control group; CFA, complete Freund adjuvant-treated group; CFA + TRM, complete Freund adjuvant and tramadol-treated group; CFA + LEV, complete Freund adjuvant and levetiracetam-treated group; CFA + CET, complete Freund adjuvant and cetirizine-treated group; ** *p* < 0.01 vs. CTL; ^#^ *p* < 0.05 vs. CFA.

**Table 3:** Thermal pain threshold for tested groups.

Rat	CTL			CFA			CFA + TRM			CFA + LEV			CFA + CET		
	Day 0	Day 7	Day 14	Day 0	Day 7	Day 14	Day 0	Day 7	Day 14	Day 0	Day 7	Day 14	Day 0	Day 7	Day 14
Mean ± S.E.M	5.35 ± 0.28	5.63 ± 0.27	6.11 ± 0.59	5.45 ± 0.42	4.93 ± 0.63	4.14 ± 0.32	6.2 ± 0.43	8.82 ± 0.97	7.29 ± 0.63	5.1 ± 0.34	6.22 ± 0.15	7.03 ± 0.87	5.82 ± 0.54	5.11 ± 0.30	5.89 ± 0.55
% variation vs. baseline		5.23	14.21		−9.54	−24.04		42.26	17.58		13.02	−6.43		15.26	25.44
% variation vs. control					−14.77	−38.24 *		37.03	3.38		7.78	−20.64		10.03	11.23
% variation vs. CFA								40.97 ^#^	41.62 ^##^		11.74	17.61		13.98	35.27 ^#^

CTL, control group; CFA, complete Freund adjuvant-treated group; CFA + TRM, complete Freund adjuvant and tramadol-treated group; CFA + LEV, complete Freund adjuvant and levetiracetam-treated group; CFA + CET, complete Freund adjuvant and cetirizine-treated group; * *p* < 0.05 vs. CTL; ^#^ *p* < 0.05, ^##^ *p* < 0.01 vs. CFA.

**Table 4:** Histopathological results—degree of inflammation.

Rat	CTL	CFA	CFA + DEXA	CFA + LEV	CFA + CET
1	0	2	1	3	2
2	0	2	1	2	2
3	0	2	1	2	2
4	0	3	1	2	2
5	0	2	1	2	2
6			1		2
Mean ± S.E.M	0	2.20 ± 0.2	1	2.20 ± 0.2	2
ANOVA F_(5, 32)_	47.93				
ANOVA *p*	<0.0001				
post-hoc	-	***	^###^	-	-

CTL, control group; CFA, complete Freund adjuvant-treated group; CFA + DEXA, complete Freund adjuvant and dexamethasone-treated group; CFA + LEV, complete Freund adjuvant and levetiracetam-treated group; CFA + CET, complete Freund adjuvant and cetirizine-treated group; S.E.M, standard error of the mean; ANOVA, analysis of variance. *** *p* < 0.001 vs. CTL; ^###^ *p* < 0.001 vs. CFA.

**Table 5:** Frequent side effects associated with levetiracetam and cetirizine use in patients.

Drug	Side Effects
Levetiracetam	Upper respiratory tract or urinary tract infections, somnolence, dizziness, behavioral effects such as depression, anxiety, aggression, nervousness, agitation, irritability, headache, asthenia, accidental injury, flu syndrome, nausea and diarrhea [8]
Cetirizine	Dry mouth, somnolence and fatigue [9]

## Data Availability

The data that support the findings of this study are available on request from the corresponding author, D.P.M.

## References

[1] Mobasheri A., Batt M. (2016). An update on the pathophysiology of osteoarthritis. Ann. Phys. Rehabil. Med..

[2] Leifer V.P., Katz J.N., Losina E. (2022). The burden of OA-health services and economics. Osteoarthr. Cartil..

[3] Conaghan P.G., Cook A.D., Hamilton J.A., Tak P.P. (2019). Therapeutic options for targeting inflammatory osteoarthritis pain. Nat. Rev. Rheumatol..

[4] Guindon J., Hohmann A. (2009). The Endocannabinoid System and Pain. CNS Neurol. Disord.—Drug Targets.

[5] Sugiura T. (2009). Physiological roles of 2-arachidonoylglycerol, an endogenous cannabinoid receptor ligand. BioFactors.

[6] Sugiura T., Kishimoto S., Oka S., Gokoh M. (2006). Biochemistry, pharmacology and physiology of 2-arachidonoylglycerol, an endogenous cannabinoid receptor ligand. Prog. Lipid Res..

[7] Chicca A., Marazzi J., Gertsch J. (2012). The antinociceptive triterpene β-amyrin inhibits 2-arachidonoylglycerol (2-AG) hydrolysis without directly targeting cannabinoid receptors. Br. J. Pharmacol..

[8] Mbizvo G.K., Dixon P., Hutton J.L., Marson A.G. (2014). The adverse effects profile of levetiracetam in epilepsy: A more detailed look. Int. J. Neurosci..

[9] Portnoy J.M., Dinakar C. (2004). Review of cetirizine hydrochloride for the treatment of allergic disorders. Expert Opin. Pharmacother..

[10] Zanfirescu A., Ungurianu A., Mihai D.P., Radulescu D., Nitulescu G.M. (2021). Targeting monoacylglycerol lipase in pursuit of therapies for neurological and neurodegenerative diseases. Molecules.

[11] Bononi G., Poli G., Rizzolio F., Tuccinardi T., Macchia M., Minutolo F., Granchi C. (2021). An updated patent review of monoacylglycerol lipase (MAGL) inhibitors (2018–present). Expert Opin. Ther. Pat..

[12] Priya M., Sathya N.V., Satyajit M., Jamuna R.R. (2013). Screening of cetirizine for analgesic activity in mice. Int. J. Basic Clin. Pharmacol..

[13] Ashmawi H.A., Braun L.M., Sousa A.M., Posso I.d.P. (2009). Analgesic Effects Of H1 Receptor Antagonists In The Rat Model Of Formalin-Induced Pain. Brazilian J. Anesthesiol..

[14] Khalilzadeh E., Azarpey F., Hazrati R. (2017). The effect of histamine h1 receptor antagonists on the morphine-induced antinociception in the acute trigeminal model of nociception in rats. Asian J. Pharm. Clin. Res..

[15] Hunskaar S., Hole K. (1987). The formalin test in mice: Dissociation between inflammatory and non-inflammatory pain. Pain.

[16] Stepanović-Petrović R.M., Micov A.M., Tomić M.A., Ugrešić N.D. (2012). The Local Peripheral Antihyperalgesic Effect of Levetiracetam and Its Mechanism of Action in an Inflammatory Pain Model. Anesth. Analg..

[17] Wiffen P.J., Derry S., Moore R.A., Lunn M.P.T., Derry S. (2014). Levetiracetam for Neuropathic Pain in Adults. Cochrane Database of Systematic Reviews.

[18] Asgharpour-Masouleh N., Rezayof A., Alijanpour S., Delphi L. (2023). Pharmacological activation of mediodorsal thalamic GABA-A receptors modulates morphine/cetirizine-induced changes in the prefrontal cortical GFAP expression in a rat model of neuropathic pain. Behav. Brain Res..

[19] Tomić M.A., Micov A.M., Stepanović-Petrović R.M. (2013). Levetiracetam interacts synergistically with nonsteroidal analgesics and caffeine to produce antihyperalgesia in rats. J. Pain.

[20] Sakin Y.S., Dogrul A., Ilkaya F., Seyrek M., Ulas U.H., Gulsen M., Bagci S. (2015). The effect of FAAH, MAGL, and Dual FAAH/MAGL inhibition on inflammatory and colorectal distension-induced visceral pain models in Rodents. Neurogastroenterol. Motil..

[21] Ignatowska-Jankowska B.M., Ghosh S., Crowe M.S., Kinsey S.G., Niphakis M.J., Abdullah R.A., Tao Q., O’Neal S.T., Walentiny D.M., Wiley J.L. (2014). In vivo characterization of the highly selective monoacylglycerol lipase inhibitor KML29: Antinociceptive activity without cannabimimetic side effects. Br. J. Pharmacol..

[22] Ghosh S., Wise L.E., Chen Y., Gujjar R., Mahadevan A., Cravatt B.F., Lichtman A.H. (2013). The monoacylglycerol lipase inhibitor JZL184 suppresses inflammatory pain in the mouse carrageenan model. Life Sci..

[23] Brindisi M., Maramai S., Gemma S., Brogi S., Grillo A., Di Cesare Mannelli L., Gabellieri E., Lamponi S., Saponara S., Gorelli B. (2016). Development and Pharmacological Characterization of Selective Blockers of 2-Arachidonoyl Glycerol Degradation with Efficacy in Rodent Models of Multiple Sclerosis and Pain. J. Med. Chem..

[24] Smith F.M., Haskelberg H., Tracey D.J., Moalem-Taylor G. (2007). Role of histamine H3 and H4 receptors in mechanical hyperalgesia following peripheral nerve injury. Neuroimmunomodulation.

[25] Obara I., Telezhkin V., Alrashdi I., Chazot P.L. (2020). Histamine, histamine receptors, and neuropathic pain relief. Br. J. Pharmacol..

[26] Tetlow L.C., Woolley D.E. (2004). Effect of histamine on the production of matrix metalloproteinases-1, -3, -8 and -13, and TNF? and PGE2 by human articular chondrocytes and synovial fibroblasts in vitro: A comparative study. Virchows Arch..

[27] Tetlow L.C., Woolley D.E. (2002). Histamine stimulates matrix metalloproteinase-3 and -13 production by human articular chondrocytes in vitro. Ann. Rheum. Dis..

[28] Yoshida H., Nagaoka A., Kusaka-Kikushima A., Tobiishi M., Kawabata K., Sayo T., Sakai S., Sugiyama Y., Enomoto H., Okada Y. (2013). KIAA1199, a deafness gene of unknown function, is a new hyaluronan binding protein involved in hyaluronan depolymerization. Proc. Natl. Acad. Sci. USA.

[29] Al-Romaiyan A., Masocha W. (2022). Pristimerin, a triterpene that inhibits monoacylglycerol lipase activity, prevents the development of paclitaxel-induced allodynia in mice. Front. Pharmacol..

[30] Jaiswal S., Ayyannan S.R. (2021). Anticancer Potential of Small-Molecule Inhibitors of Fatty Acid Amide Hydrolase and Monoacylglycerol Lipase. ChemMedChem.

[31] Puris E., Petralla S., Auriola S., Kidron H., Fricker G., Gynther M. (2023). Monoacylglycerol Lipase Inhibitor JJKK048 Ameliorates ABCG2 Transporter-Mediated Regorafenib Resistance Induced by Hypoxia in Triple Negative Breast Cancer Cells. J. Pharm. Sci..

[32] Wittig F., Henkel L., Prüser J.L., Merkord J., Ramer R., Hinz B. (2023). Inhibition of Monoacylglycerol Lipase Decreases Angiogenic Features of Endothelial Cells via Release of Tissue Inhibitor of Metalloproteinase-1 from Lung Cancer Cells. Cells.

[33] Luo S., Li H., Liu J., Xie X., Wan Z., Wang Y., Zhao Z., Wu X., Li X., Yang M. (2020). Andrographolide ameliorates oxidative stress, inflammation and histological outcome in complete Freund’s adjuvant-induced arthritis. Chem. Biol. Interact..

[34] Deuis J.R., Dvorakova L.S., Vetter I. (2017). Methods used to evaluate pain behaviors in rodents. Front. Mol. Neurosci..

[35] Falk S., Ipsen D.H., Appel C.K., Ugarak A., Durup D., Dickenson A.H., Heegaard A.M. (2015). Randall Selitto pressure algometry for assessment of bone-related pain in rats. Eur. J. Pain.

[36] Kayser V. (2013). Randall-Selitto Paw Pressure Test. Encyclopedia of Pain.

[37] Zanfirescu A., Cristea A.N., Nitulescu G.M., Velescu B.S., Gradinaru D. (2018). Chronic monosodium glutamate administration induced hyperalgesia in mice. Nutrients.

[38] Fierascu R.C., Georgiev M.I., Fierascu I., Ungureanu C., Avramescu S.M., Ortan A., Georgescu M.I., Sutan A.N., Zanfirescu A., Dinu-Pirvu C.E. (2018). Mitodepressive, antioxidant, antifungal and anti-inflammatory effects of wild-growing Romanian native *Arctium lappa* L. (Asteraceae) and Veronica persica Poiret (Plantaginaceae). Food Chem. Toxicol..

[39] Hayer S., Vervoordeldonk M.J., Denis M.C., Armaka M., Hoffmann M., Bäcklund J., Nandakumar K.S., Niederreiter B., Geka C., Fischer A. (2021). SMASH recommendations for standardised microscopic arthritis scoring of histological sections from inflammatory arthritis animal models. Ann. Rheum. Dis..

[40] Aida J., Fushimi M., Kusumoto T., Sugiyama H., Arimura N., Ikeda S., Sasaki M., Sogabe S., Aoyama K., Koike T. (2018). Design, Synthesis, and Evaluation of Piperazinyl Pyrrolidin-2-ones as a Novel Series of Reversible Monoacylglycerol Lipase Inhibitors. J. Med. Chem..

[41] Land H., Humble M.S. (2018). YASARA: A Tool to Obtain Structural Guidance in Biocatalytic Investigations. Protein Engineering: Methods and Protocols.

[42] Zanfirescu A., Nitulescu G., Mihai D.P., Nitulescu G.M. (2021). Identifying FAAH Inhibitors as New Therapeutic Options for the Treatment of Chronic Pain through Drug Repurposing. Pharmaceuticals.

[43] Trott O., Olson A.J. (2010). AutoDock Vina: Improving the speed and accuracy of docking with a new scoring function, efficient optimization, and multithreading. J. Comput. Chem..

